# Genotoxicity of fine and coarse fraction ambient particulate matter in immortalised normal (TT1) and cancer‐derived (A549) alveolar epithelial cells

**DOI:** 10.1002/em.22166

**Published:** 2018-01-25

**Authors:** Ian W.H. Jarvis, Zachary Enlo‐Scott, Eszter Nagy, Ian S. Mudway, Teresa D. Tetley, Volker M. Arlt, David H. Phillips, B. Gollapudi

**Affiliations:** ^1^ Department of Analytical, Environmental and Forensic Sciences, MRC‐PHE Centre for Environment and Health King's College London London United Kingdom; ^2^ NIHR HPRU in Health Impact of Environmental Hazards at King's College London in Partnership with Public Health England in collaboration with Imperial College London London United Kingdom; ^3^ Lung Cell Biology, Airways Disease, National Heart & Lung Institute, Imperial College London London United Kingdom

**Keywords:** air pollution, alveolar epithelium, DNA damage response, genotoxicity, particulate matter, polycyclic aromatic hydrocarbons

## Abstract

Human exposure to airborne particulate matter (PM) is associated with adverse cardiopulmonary health effects, including lung cancer. Ambient PM represents a heterogeneous mixture of chemical classes including transition metals, polycyclic aromatic hydrocarbons (PAHs) and their derivatives such as nitro‐PAHs, many of which are classified as putative carcinogens. As the primary site of human exposure to PM is the lungs, we investigated the response of two alveolar epithelial cell lines, the tumour‐derived A549 and newly described TT1 cells, to fine and coarse PM collected from background and roadside locations. We show that coarse PM elicits a genotoxic response in the TT1 cells, with the strongest signal associated with the background sample. This response could be recapitulated using the organic extract derived from this sample. No responses were observed in PM‐challenged A549 cells. Fine PM failed to elicit a genotoxic response in either cell line despite the higher PAH concentrations within this fraction. Consistent with the lack of a simplistic association between PM PAH content and the observed genotoxic response, TT1 cells treated with benzo[*a*]pyrene (BaP) demonstrated no increase in the selected markers. In contrast, a pattern of response was observed in TT1 cells challenged with 3‐nitrobenzanthrone (3‐NBA) similar to that with coarse PM. Together, these data illustrated the suitability of the TT1 cell line for assessing PM‐induced genotoxicity and challenge the contention that fine roadside PM poses the higher cancer risk. Furthermore, the response to 3‐NBA and not BaP suggests a major contribution of nitro‐PAHs to the overall toxicity of PM. Environ. Mol. Mutagen. 59:290–301, 2018. © 2018 The Authors Environmental and Molecular Mutagenesis published by Wiley Periodicals, Inc. on behalf of Environmental Mutagen Society

**Abbreviations**
3‐NBA: 3‐nitrobenzanthrone
BaP: benzo[*a*]pyrene
BPDE: benzo[a]pyrene diol‐epoxide
CYP: cytochrome P450
GHM: Großhadern Hospital, Munich; IL‐8, interleukin‐8
NQO1: NAD(P)H: [quinone oxidoreductase] 1
OM: Ostbahnhof, Munich
OP^AA^: ascorbate dependent oxidative potential
OP^GSH^: glutathione dependent oxidative potential
PAHs: polycyclic aromatic hydrocarbons
PM: particulate matter

## INTRODUCTION

According to the World Health Organization, air pollution contributes to 12.5% of world‐wide deaths [World Health Organisation, 2014], making it a major contributor to the global burden of disease. Particulate matter (PM), consisting of extremely small and chemically complex particles and liquid droplets in the air, represents a significant component of the pollution mixture and has been shown to have significant acute and long‐term health effects in exposed populations [World Health Organisation, 2013]. In urban areas, primary emissions of particulate matter derived from traffic, domestic heating, and industrial sources have been demonstrated to contribute significantly to observed health effects [Rohr and McDonald, 2016]. Recently, there has been an increased focus on diesel tail pipe emissions, with epidemiological evidence linking markers of diesel exhaust (elemental and black carbon) to a diverse range of health endpoints, ranging from a worsening of respiratory symptoms [Samoli et al., 2016] to premature death [Atkinson et al., 2016]. In addition the incomplete pyrolysis of carbon‐containing compounds, particularly from diesel fuel, is a major source of polycyclic aromatic hydrocarbons (PAHs) and their derivatives, including nitrated PAHs (nitro‐PAHs), in ambient PM [Bamford et al., 2003]. Both PAHs and nitro‐PAHs have attracted considerable interest with regards to their effects on human health given their ability, following intracellular metabolism, to bind to DNA, and induce mutations [Baird et al., 2005; Nagy et al., 2007]. Recently, the International Agency for Research on Cancer (IARC) classified outdoor air pollution and diesel engine emissions as carcinogenic to humans [IARC, 2013, 2016].

The primary route of human exposure to PM‐associated PAHs and nitro‐PAHs is through inhalation and they have been argued to represent a major underlying cause of respiratory disease [World Health Organisation, 2005; IARC, 2010, 2016]. The conducting airways (from the trachea to the terminal bronchioles) are multicellular by nature, whereas in contrast, there are only two types of cells lining the alveoli. Type I alveolar cells are squamous and contribute between 90% and 95% of the alveolar surface in adult lungs. They represent the gas exchange interface between the lungs and the circulatory system. As they are unable to replicate, Type I alveolar cells are particularly sensitive to damaging insults. Type II alveolar cells are cuboidal, and whilst they only contribute between 3% and 5% of the alveolar surface, they account for around two thirds of the total alveolar epithelial cells. Type II cells are primarily responsible for secretion of pulmonary surfactant and play an important immune‐regulatory role in the lung; as such they have been labelled defenders of the alveolus [Fehrenbach, 2001]. Type II cells are capable of cell division and act as the main progenitor cells during repair of alveolar damage, functioning as self‐renewing cells, and precursors of Type I cells [Barkauskas et al., 2013].

Given the evidence of human exposure to PAHs in urban [Zhou et al., 2016] and occupational settings [Wang et al., 2015], studies on lung cell models are highly relevant for understanding the detrimental effects of such exposures on human health. The A549 cell line is a human‐derived lung adenocarcinoma cell line with a morphology and basic cellular functions similar to human alveolar Type II (ATII) epithelial cells [Foster et al., 1998]. However, the evidence suggests that A549 cells have a distinctly different architecture and barrier properties to ATII cells, do not transition to an ATI phenotype like primary ATII cells, and have inconsistencies in expression of ATII cell markers, which has led some researchers to question their suitability as an in vitro model [Swain et al., 2010]. More recently, the first human alveolar epithelial Type I‐like cell line, TT1, was reported, derived from immortalisation of primary human ATII cells (progenitors of ATI cells) [Kemp et al., 2008]. TT1 cells demonstrate a more ATI‐like phenotype and have been described as a reasonable in vitro model for ATI cells [Swain et al., 2010].

In the present study, we investigated the genotoxic response of TT1 and A549 cells to coarse (2.5–10 µm) and fine (0.1–2.5 µm) PM collected at background and roadside locations. Contrary to our expectations, we found that the coarse PM sample from the background location yielded the strongest genotoxic response. No response was seen following incubation with the fine PM samples from either location. The response to coarse PM was replicated using organic extracts alone. To elucidate further, we studied the response of both cell lines to 3‐nitrobenzanthrone (3‐NBA), a highly mutagenic nitro‐PAH and suspected human lung carcinogen [Arlt, 2005], and benzo[*a*]pyrene (BaP), a widely studied PAH and, to date, the only PAH classified by IARC as carcinogenic to humans (Group 1) [IARC, 2010]. We demonstrated a stronger genotoxic following exposure to 3‐NBA than BaP, potentially suggesting a more prominent role for nitro‐PAHs.

## MATERIALS AND METHODS

### Cell Culture

TT1 cells were cultured in DCCM‐1 medium (Biological Industries, UK) containing 10% foetal bovine serum (Invitrogen, UK), 2 mM l‐glutamine (Invitrogen, UK) and penicillin (100 units/ml) and streptomycin (0.1 mg/ml) (Invitrogen, UK) as described previously [Kemp et al., 2008]. A549 cells were obtained from the American Type Culture Collection (ATCC, MD) and cultured in DMEM supplemented with 10% foetal calf serum and penicillin (100 units/ml) and streptomycin (0.1 mg/ml). Cells were seeded at a density of 2 × 10^4^/cm^2^ and incubated at 37°C and 5% CO_2_ for 24 hr prior to exposure.

### Particulate Matter Samples and Cell Exposure

The particulate matter (PM) samples used in this study are derived from an archive originally collected as part of the HEPMEAP project [Bloemen et al., 2005; Sandstrom et al., 2005]. Sets of parallel fine and coarse PM samples (labelled _F and _C, respectively) were collected at two urban sites in Munich, Germany; at a busy roadside site (Central Munich Ost Bahnhof, collected 18th November–2nd December 2002, labelled ‘RS’) and a background location, situated away from traffic (Munich Großhadern Hospital, collected 17th–31st May 2002, labelled ‘BG’). Briefly, PM samples were collected on polyurethane foam by impaction using a high‐volume cascade impactor at a flow rate of 900 l/min. The impactor cut points were 9.9, 2.46, and 0.12 µm. PM was extracted by sonicating in methanol, and then concentrated in methanol by rotary evaporation before incubation overnight at 30°C. PM concentrations were determined by measuring the mass of the PM‐containing tube that had been pre‐measured prior to use. All samples were stored at −70°C as dried extracts until required. PM samples were analysed for a number of different chemicals including PAHs and metals/metalloids. Detailed information on elemental and PAH composition on these samples has been published previously [Bloemen et al., 2005]; detailed PAH and metal compositions are given in Supporting Information Tables 1 and 2, respectively, and the relative contribution of IARC classified PAHs, metals, and metalloids are illustrated in Supporting Information Figure 1 (panels A and B). At the time of collection, analysis of nitro‐PAHs was not available. For cell exposures, PM was suspended in DMSO and sonicated for 30 min prior to use; stocks were stored at −20°C. Where indicated, PM was centrifuged at 10,000*g* for 60 sec prior to exposure.

### Test Compounds and Cell Exposure

Benzo[*a*]pyrene (BaP, CAS number 50‐32‐8; purity ≥96%) was obtained from Sigma Aldrich (United Kingdom). 3‐Nitrobenzanthrone (3‐NBA, CAS number 17117‐34‐9) was prepared as previously reported [Arlt et al., 2002]. Stocks of BaP and 3‐NBA were dissolved in DMSO and stored at –20°C. Test compounds were diluted in fresh medium to final concentration and then added to the cells (seeding medium was aspirated immediately prior). Cells were exposed to DMSO (solvent control) or test compounds for 24 hr. The DMSO concentration was always kept at < 0.5% of the total culture medium volume.

### Analysis of Cell Viability

Cell viability was assessed using the AlamarBlue assay which is based on the reduction of resazurin to resorufin in metabolically active cells. Following exposure, cells were incubated with a 10% solution of AlamarBlue reagent (Invitrogen, UK) in culture medium at 37°C and 5% CO_2_ for 60 min. After incubation, the resulting solution in each well was diluted 10 × in PBS on a 96‐well plate (in triplicate per experiment) and fluorescence measured at excitation/emission wavelengths 530/590 nm using a Synergy HT plate reader (Biotek, UK). Results are expressed as percentage cell viability versus DMSO control.

### Oxidative Potential of PM Samples

The oxidative activity of all four PM samples was assessed by quantifying their capacity to deplete ascorbate (AA) and glutathione (GSH) from a composite antioxidant solution, reflecting their in vivo concentrations in human respiratory tract lining fluids (RTLF) over a 4‐hr incubation (37°C, pH 7.0), at final concentration of 50 μg/ml. AA and GSH‐dependent oxidative potential (OP) was then determined as the % loss over the 4‐hr incubation, relative to a particle free control, per μg of sample: OP^AA^ and OP^GSH^/μg, respectively. Details of this methodology and the derivation of the OP metrics have been described in detail previously [Godri et al., 2010].

### Enzyme‐Linked Immunosorbent Assay (ELISA) for Interleukin 8 (IL‐8)

Conditioned medium was collected following exposure and assayed for IL‐8 content using Human Quantikine CXCL8/IL‐8 (D8000C) kits (R&D Systems, Bio‐Techne, UK) according to the manufacturer's guidelines. Prior to assaying, medium samples were centrifuged at 10,000*g* for 10 min and supernatants used for the ELISA. Absorbance was measured at 450 nm and 570 nm for background correction using a Synergy HT plate reader (Biotek, UK).

### Protein Analysis by Western Blotting

Whole cell lysates were prepared in cold lysis buffer (62.5 mM Tris pH 6.8, 1 mM EDTA pH 8.0, 2% SDS and 10% glycerol) containing Halt Protease and Phosphatase Inhibitor Cocktail (Thermo Scientific, UK). Protein content was measured in sonicated samples using the BCA Protein Assay (Thermo Scientific, UK) according to the manufacturer's instructions. Equal amounts of protein were separated by SDS–PAGE using 4–12% bis‐tris gels (Invitrogen, UK) in MES buffer (Invitrogen, UK). Separated proteins were transferred to a nitrocellulose membrane (Bio‐Rad, UK) by wet electro‐blotting. Non‐specific antibody binding was reduced by incubating membranes in 5% non‐fat dry milk in TBS with 0.1% Tween‐20 (TBS‐T). Membranes were incubated overnight at 4°C with primary antibodies prepared in 5% milk/TBS‐T. Cell Signaling Technology (Beverly, MA) provided anti‐Chk1 phosphorylated at Ser317 (pChk1, #2348) and anti‐H2AX phosphorylated at Ser139 (pH2AX, #9718) antibodies. Anti‐Cdk2 was obtained from Santa Cruz Biotechnology (sc‐163, Santa Cruz, CA) and included in all experiments as a loading control. After washing, membranes were incubated with secondary antibody prepared in 5% milk/TBS‐T for 60 min at room temperature. Immun‐Star goat anti‐rabbit HRP conjugated secondary antibody was obtained from Bio‐Rad (1705046, Bio‐Rad, UK). Signals were detected using Amersham ECL Western Blotting Detection Reagent (GE Healthcare Lifescience, UK). Experiments were performed at least three times and analysed separately. Densitometric analysis was performed using ImageJ software version 1.48v (National Institute of Health). Results are expressed as fold increases normalised to control levels.

### Analysis of DNA Damage by Comet Assay

The alkaline comet assay was performed as described previously [Nagy et al., 2005], with minor modifications. In brief, three‐window diagnostic slides (Thermo Fisher Scientific Gerhard Menzel B.V. & Co, Germany) were coated with 0.75% (*w*/*v*) low gelling temperature agarose (A9414, Sigma‐Aldrich, UK) per window and left to dry overnight at room temperature. Cells were collected following exposure, resuspended in 0.75% agarose and applied per window of the diagnostic slide. Following incubation in cold lysis buffer (2.5 M sodium chloride, 10 mM Tris, and 0.1M EDTA, pH 10 with 1% (*w*/*v*) Triton X‐100) for 60 min on ice and, slides were incubated in cold alkaline unwinding solution (0.3 M sodium hydroxide, 1 mM EDTA). Electrophoresis was performed in the same buffer and temperature for 20 min at 21 V. Subsequently, slides were neutralized in 0.4 M Tris‐HCl (pH 7.4) and then fixed in 100% methanol for 5 min before air‐drying overnight in the dark. Nuclei were stained with ethidium bromide (2 µg/ml in water) and washed in deionized water. A total of 50 comets were scored using a Leica DMLB fluorescent microscope and Comet IV capture system (Perceptive Instruments, UK). Tail intensity (% tail DNA), defined as the percentage of DNA migrated from the head of the comet into the tail, was used as a measure of DNA damage.

### Detection of DNA Adducts by ^32^P‐Postlabelling Analysis

DNA was isolated from cells following exposure using a standard phenol/chloroform extraction method. DNA adduct formation was analysed by the nuclease P1 digestion and butanol extraction enrichment version of the ^32^P‐postlabelling method as described previously [Phillips and Arlt, 2014; Krais et al., 2016]. Briefly, DNA samples (4 μg) were digested with micrococcal nuclease (288 mU; Sigma Aldrich, UK) and calf spleen phosphodiesterase (1.2 mU; MP Biomedical, France) and then enriched and labelled as reported. Resolution of ^32^P‐labelled adducts was performed by polyethyleneimine(PEI)‐cellulose thin‐layer chromatography (TLC) using the following chromatographic conditions: D1: 1.0 M sodium phosphate, pH 6.0; D3: 4.0 M lithium formate, 7.0 M urea, pH 3.5; D4: 0.8 M lithium chloride, 0.5 M Tris, 8.5 M urea, pH 8.0. After chromatography, TLC plates were scanned using a Packard Instant Imager (Downers Grove, IL), and DNA adduct levels (RAL, relative adduct labelling) were calculated from the adduct counts per minute (cpm), the specific activity of [γ‐^32^P]ATP and the amount of DNA (pmol) used. Results were expressed as relative DNA adducts levels/10^8^ nucleotides. An external benzo[a]pyrene diol‐epoxide(BPDE)‐DNA standard [Phillips and Castegnaro, 1999] was employed for identification of adducts in experimental samples. 3‐NBA‐derived DNA adducts were identified as reported [Arlt et al., 2001; Arlt et al., 2006].

### RNA Extraction and Real‐Time RT‐PCR

RNA was extracted using the RNEasy mini kit (Qiagen, Hilden, Germany) and 1 µg was reverse transcribed into cDNA with the high‐capacity RNA to cDNA kit (Applied Biosystems, CA) according to manufacturer guidelines. Subsequently, analysis of gene expression was performed in triplicates on each plate using the TaqMan Gene Expression Master Mix (Applied Biosystems, CA) with detection on an Applied Biosystems 7900HT Fast Real‐Time PCR system (Applied Biosystems, CA). The cycling parameters used for the real‐time RT‐PCR were 50°C for 2 min, 95°C for 10 min, and then 40 cycles at 95°C for 15 sec and 60°C for 60 sec. Primers and probes were designed using the Universal Probe Library (Roche, Switzerland). Primer sequences were as follows: *CYP1A1* forward TCCAAGAGTCCACCCTTCC and reverse AAGCATGATCAGTGTAGGGATCT, *NQO1* forward CAGCTCACCGAGAGCCTAGT and reverse GAGTGAGCCAGTACGATCAGTG, and *GAPDH* forward AGCCACATCGCTCAGACAC and reverse AATACGACCAAATCCGTTGACT. Quantification of relative gene expression was based on the comparative threshold cycle method (2^−ΔΔCt^).

### Statistical Analysis

All data are presented as means ± standard deviation (SD) and are representative of at least three independent experiments. Statistical analysis was performed on the raw data (i.e. non‐normalized). One‐way repeated measures ANOVA with Tukey's post‐hoc test was used to determine statistical significance (*P* < 0.05).

## RESULTS

### Genotoxic Effects of PM Mixtures

**Figure 1:**
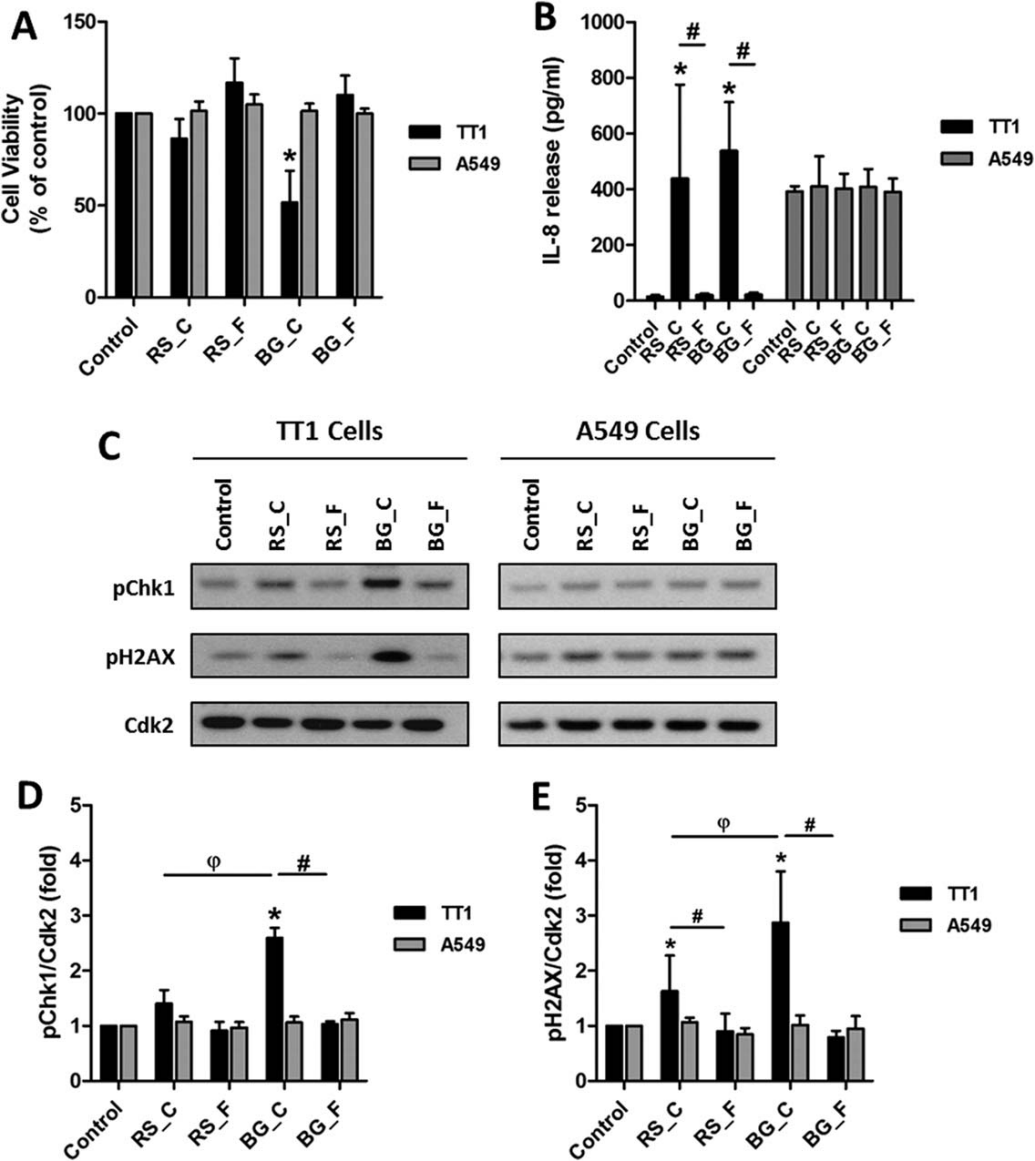
Response of TT1 and A549 cells exposed to coarse and fine PM samples. Cells were exposed to DMSO control or 10 µg/ml of PM in DMSO. PM samples are from roadside (RS) and background (BG) locations; coarse and fine fractions are indicated by _C and _F, respectively. **A**: Cell viability as assessed by AlamarBlue assay. **B**: Levels of IL‐8 in culture supernatants as determined by ELISA. **C**: Western blot analysis of phosphorylation of Chk1 and H2AX; Cdk2 was included as a loading control. **D** and **E**: Densitometric analysis of levels of phosphorylated Chk1 and H2AX as assessed by Western blotting. Data are normalised against control levels and hence no error bars are presented in the control samples. Experiments were performed at least in triplicate and data points represent the mean values ± SD. * *P* < 0.05 versus control, # *P* < 0.05 between locations and φ *P* < 0.05 between coarse fractions as determined by one‐way repeated measures ANOVA with Tukey's post hoc test.

TT1 and A549 cells were exposed to coarse and fine PM samples from roadside and background locations at concentrations of 10 µg/ml in DMSO for 24 hr. A significant cytotoxic response was seen in TT1 cells exposed to the coarse background PM (BG_C) showing approximately 50% decrease in cell viability compared to control cells (Fig. 1A). A small but non‐statistically significant decrease in viability was seen in TT1 cells exposed to the coarse roadside PM (RS_C); no effect on viability was seen in TT1 cells exposed to fine PM or in any of the A549 cell exposures (Fig. 1A). As ambient PM has been shown to increase expression of IL‐8 mRNA [Silbajoris et al., 2011], we examined IL‐8 concentrations in media of cells exposed to PM samples. With TT1 cells, significant increases in IL‐8 were seen after exposure to coarse PM from both background and roadside locations compared to unexposed cells; no differences compared to controls were seen after exposure to fine PM. Comparable levels of IL‐8 were detected after exposure of TT1 cells to the coarse PM samples despite the differences in cell viability. In contrast, no changes in IL‐8 levels were seen in A549 cells exposed to coarse or fine PM (Fig. 1B). Of note, background levels of IL‐8 release from A549 cells were approximately 27‐times higher than TT1 cells; the reason for this is unclear.

DNA damage and the resulting activation of damage signalling are known causes of toxicity of PM and associated chemicals. We chose two markers of DNA damage signalling activation, phosphorylation of Checkpoint kinase 1 at Ser317 (pChk1) and H2A histone family member X at Ser139 (pH2AX), as both have previously been shown to be activated in response to PAH and PM exposures [Niziolek‐Kierecka et al., 2012; Jarvis et al., 2013]. In TT1 cells, levels of pChk1 were significantly increased in cells exposed to coarse PM from the background site (BG_C); this was significantly different to unexposed cells, to cells exposed to fine PM from the same location (BG_F), and to cells exposed to coarse PM from the roadside site (RS_C) (Figs. 1C and 1D). Levels of pH2AX were significantly increased in TT1 cells exposed to coarse PM from both the background (BG_C) and roadside (RS_C) locations compared to unexposed cells and to cells exposed to fine PM from their respective sites (Figs. 1C and 1E). Levels of pH2AX were also significantly higher in TT1 cells exposed to coarse PM from the background site compared to the roadside site. In contrast, no increase in pChk1 or pH2AX was seen in A549 cells exposed to coarse or fine PM from either site. For this reason, from this point experiments were continued using only the TT1 cell line.

**Figure 2:**
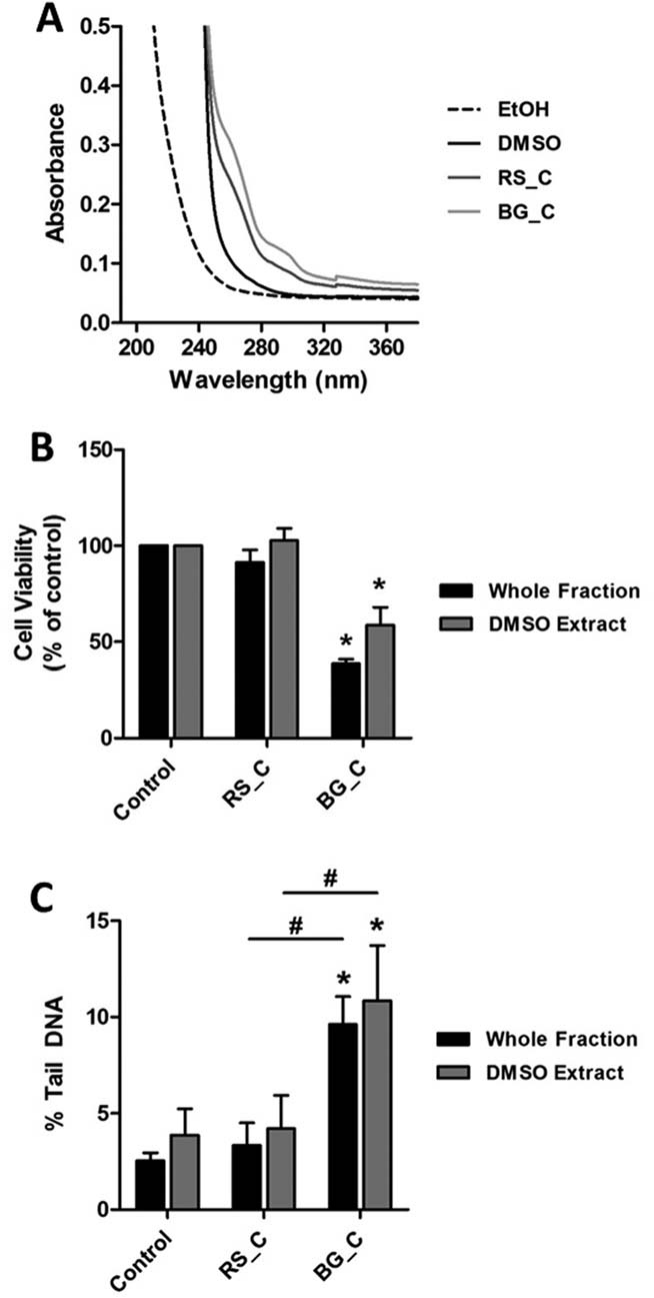
Response of TT1 cells exposed to whole fractions and centrifuged DMSO extracts of coarse PM samples. Cells were exposed to DMSO control or 10 µg/ml of coarse fraction PM in DMSO. Whole fraction label indicates PM suspended in DMSO; the DMSO extract label indicates cells were exposed to the supernatant of the whole fraction after centrifugation (10,000 × g for 60 sec). **A**: UV absorbance of DMSO and PM samples; EtOH was used as a vehicle. **B**: Cell viability as assessed by AlamarBlue assay; data are normalised to control levels and hence no error bars are presented in the controls. **C**: DNA damage was assessed by alkaline comet assay. Experiments were performed at least in triplicate and data points represent the mean values ± standard deviation. * *P* < 0.05 versus control and # *P* < 0.05 between exposures as determined by one‐way repeated measures ANOVA with Tukey's post hoc test.

To determine whether the effect of the coarse PM samples could be attributed to compounds that likely desorb from particles in DMSO, TT1 cells were exposed to coarse PM suspended in DMSO and centrifuged extracts of these samples. UV‐Vis analysis of the DMSO extracts showed an increase in absorbance between 260 and 300 nm (Fig. 2A), suggesting the presence of UV absorbing compounds. Whilst the coarse PM sample from the background location (BG_C) induced a significant decrease in cell viability (Fig. 2B) and increase in DNA damage (Fig. 2C), there was no detectable difference between the whole fraction (particles suspended in DMSO) and the DMSO supernatant. Together, these data show that coarse PM induces a genotoxic response in the TT1 cell line and suggest that the observed response results from organic materials that have desorbed from the particles into the DMSO. It was notable however that whilst the concentration of detected PAHs (classified as IARC Group 1, 2A, or 2B carcinogens) was greater in the coarse PM sample from the background site compared with the equivalent roadside sample, these concentrations were significantly lower than those associated with the fine fraction collected from both sites (Supporting Information Fig. 1A). These results suggested that these PAHs within the organic fraction were unlikely to be solely responsible for the genotoxic responses observed. Similarly metal and metalloid concentrations (classified as IARC Group 1 or 2B carcinogens) across the four PM samples did not fit with the pattern of responses observed (Supporting Information Fig. 1B). The oxidative activity of the samples, especially OP^AA^/μg, was clearly enhanced at the roadside site (Supporting Information Fig. 1C) and likewise not predictive of the genotoxic responses observed.

### 3‐NBA Induces a Potent Genotoxic Response in TT1 Cells That Is Not Seen with BaP

**Figure 3:**
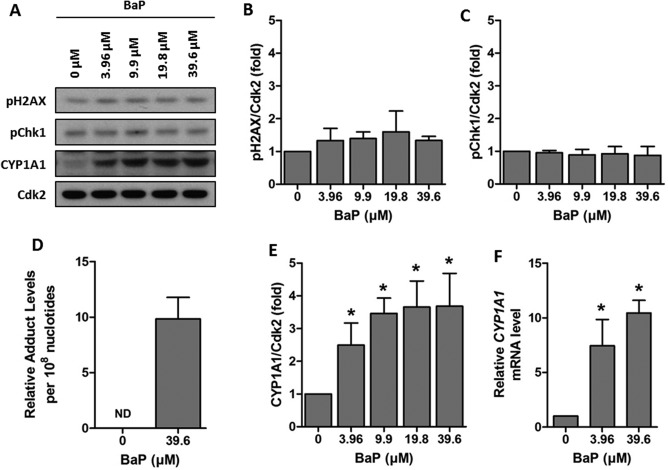
Genotoxic response of TT1 cells exposed to BaP. Cells were exposed to 0 – 39.6 µM of BaP for 24 hr. A: Representative Western blots of pH2AX, pChk1 and CYP1A1. **B** and **C**: Densitometric analysis of levels of pH2AX and pChk1 assessed by Western blotting. **D**: ^32^P‐postlabelling analysis of BaP‐DNA adducts in cells exposed to 39.6 µM BaP (ND indicates no detected levels in control cells). **E**: Densitometric analysis of levels of CYP1A1 assessed by Western blotting. **F**: Real‐time RT‐PCR analysis of *CYP1A1* mRNA. Data are normalised against control levels and hence no error bars are presented in the control samples. Experiments were performed at least in triplicate and data points represent the mean values ± SD. * *P* < 0.05 as determined by one‐way repeated measures ANOVA with Tukey's post hoc test.

**Figure 4:**
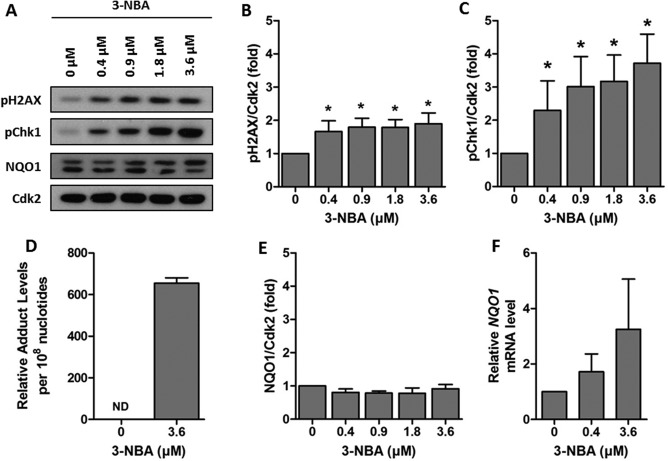
Genotoxic response of TT1 cells exposed to 3‐NBA. Cells were exposed to 0 – 3.6 µM of 3‐NBA for 24 hr. A: Representative Western blots of pH2AX, pChk1 and NQO1. **B** and **C**: Densitometric analysis of levels of pH2AX and pChk1 assessed by Western blotting. **D**: ^32^P‐postlabelling analysis of 3‐NBA‐DNA adducts in cells exposed to 3.6 µM 3‐NBA (ND indicates no detected levels in control cells). **E**: Densitometric analysis of levels of NQO1 assessed by Western blotting. **F**: Real‐time RT‐PCR analysis of *NQO1* mRNA. Data are normalised against control levels and hence no error bars are presented in the control samples. Experiments were performed at least in triplicate and data points represent the mean values ± SD. * *P* < 0.05 as determined by one‐way repeated measures ANOVA with Tukey's post hoc test.

Having shown that coarse PM suspended in DMSO and the corresponding centrifuged extract produce similar genotoxic responses in TT1 cells, we sought to clarify which organic constituents were contributing to the observed responses. We initially investigated the response of TT1 cells to BaP, the most widely studied carcinogenic PAH. At the concentrations of BaP tested (0–39.6 µM), we saw no significant cytotoxicity (Supporting Information Fig. 2A). No significant increase in either pChk1 or pH2AX levels was observed (Figs. 3A–3C) and there was only a small amount of BaP‐DNA adducts detected (9.84 ± 1.93 adducts per 10^8^ nucleotides) (Fig. 3D, Supporting Information Fig. 3A). BaP is primarily metabolised by CYP1A1, and significantly increased levels of CYP1A1 protein (Figs. 4A and 4E) and *CYP1A1* mRNA (Fig. 4F) were observed. We next investigated if this response could be attributed to nitro‐PAHs, which have been strongly associated with engine exhausts emissions [Arlt, 2005]. TT1 cells were therefore incubated with 3‐NBA, a highly mutagenic nitro‐PAH and suspected lung carcinogen. At the concentrations of 3‐NBA tested (0–3.6 µM), no significant cytotoxicity was observed (Supporting Information Fig. 2B). Exposure to 3‐NBA caused a significant increase in pChk1 and pH2AX at all concentrations tested (Figs. 4A–4C), and this increase in DNA damage signalling was associated with a high level of 3‐NBA‐DNA adducts (654.77 ± 25.73 adducts per 10^8^ nucleotides) (Fig. 4D and Supporting Information Fig. 3B). In order to react with DNA, 3‐NBA requires metabolism to the active *N*‐hydroxy‐3‐aminobenzanthrone (*N*‐OH‐3‐ABA) metabolite, primarily catalysed by nitroreductases such as NAD(P)H:quinone oxidoreductase 1 (NQO1). No significant increases in levels of NQO1 protein were detected (Figs. 3A and 3E), although a small but not statistically significant increase in *NQO1* mRNA was observed (Fig. 3F). Together these data show that 3‐NBA induces a potent genotoxic response in TT1 cells that is not associated with elevated NQO1 levels and that a nitro‐PAH can induce a strong genotoxic response in the TT1 cell line that is not seen with BaP.

## DISCUSSION

One of the most important aspects of studying human toxicity in an in vitro system is selecting appropriate models that can predict toxicity in vivo. The primary target for air pollutants is the lungs and hence that was the rationale for our selection of lung cell lines. In this study, we used two alveolar epithelial cell‐like cell lines, TT1 (Type‐1 like) and A549 (Type‐II like); both surrogate alveolar epithelial cell models. We studied first the effects of exposing them to well characterised coarse (2.5–10 µm) and fine (0.1–2.5 µm) PM from roadside and background locations. We selected activation of DNA damage signalling, indicated by phosphorylation of Chk1 and H2AX, as markers of response, based on previous studies showing these to be sensitive markers [Audebert et al., 2010; Niziolek‐Kierecka et al., 2012; Jarvis et al., 2013] with potential application for mixture‐based risk assessment [Jarvis et al., 2014]. We found that coarse PM induces a genotoxic response in the TT1 cells, but not in A549 cells, and contrary to initial expectations, this response was strongest from the background sample. We also studied secretion of IL‐8 in response to PM exposure, IL‐8 having been identified (along with IL‐6) as being secreted in response to radiation‐induced DNA damage [Rodier et al., 2009]. We then used the TT1 cell line to study the genotoxic response (activation of damage signalling and formation of DNA adducts) in response to BaP and 3‐NBA, and showed a strong genotoxic response to latter but not former. Furthermore, we show that the TT1 cell line is a valid alveolar epithelial model for studying genotoxic effects of PM and associated chemicals.

Humans are exposed to a complex mixture of different chemicals in the environment. Studying the genotoxic effects of whole mixtures is therefore more applicable to human exposure scenarios and this approach has been proposed to represent a more appropriate exposure scenario for risk assessment of human health effects of airborne pollution [United States EPA., 2000; Flowers et al., 2002; United States EPA., 2007; Backhaus et al., 2010; Jarvis et al., 2014]. However, given the ubiquitous nature of chemical mixtures, and hence the complicated interpretation of contributory effects and interactions, the study of individual compounds can also provide important contextual information. The rationale for selection of the two pollutants studied here alongside PM samples is that BaP is the most widely studied PAH and at present the only PAH classified by IARC as carcinogenic to humans [IARC, 2010], and 3‐NBA is a highly mutagenic nitro‐PAH associated with traffic emissions [Arlt, 2005] and classified by IARC as Group 2B (possibly carcinogenic to humans) [IARC, 2013].

With regards to PM exposure, it is generally considered that PM <10 µm is able to penetrate to the bronchiolar regions of the respiratory system, whereas PM <2.5 µm is considered to be able to penetrate to the gas exchange regions, the alveolar‐capillary barrier. In this study, we used PM characterised as ‘coarse’, which ranged from 2.5–10 µm in size, and ‘fine’, which ranged from 0.1–2.5 µm in size. Detailed compositional information on the samples employed in this study has been presented previously, including concentrations of metals and metalloids, PAHs, hopanes, sterenes, secondary species (nitrite and sulphate) and biological material (LPS) [Bloemen et al., 2005]. Although the coarse PM studied here would not be expected penetrate the alveolar region in vivo, its use here in an isolated in vitro system was to better understand its genotoxic profile. Studies of this nature can provide important information regarding mechanisms of actions of various compounds and mixtures and can advance our knowledge for interpreting effects in more complex systems. Nevertheless, extrapolation of the findings to ‘real‐life’ exposure scenarios is limited by several factors. Firstly, the cell models lack the multicellular nature of the respiratory system. Secondly, the PM in this scenario was suspended in DMSO due to an interest in the organic material and hence is not representative of inhalation, whereby PM is rapidly in contact with water droplets in the airways and surfactant at the lung surfaces. Finally, the concentrations used in the study are higher than humans would be exposed to under realistic exposure scenarios. For example, Rossner et al. [2016] suggested that for an ambient concentration of BaP of 0.1–10 ng/m^3^ in air this would represent femtomolar concentrations of BaP in culture medium. On the other hand, human lungs are typically chronically exposed to low levels of air pollutants, which is difficult to mimic in any cell culture models. Thus, testing acute exposures at higher concentrations may still have value to predict cellular responses and toxic mechanisms that might be the same as those seen with chronic exposures at low concentrations. The results of this study may therefore contribute to the understanding of the mechanisms of actions of PM and 3‐NBA in relation to the effects on DNA damage.

Mutations resulting from DNA damage are critical to the carcinogenic process resulting from exposure to PAHs and derivatives [Baird et al., 2005]. In response to DNA damage, cells have evolved a number of mechanisms involved in sensing the damage, arresting the cell cycle, and ultimately eliciting repair, collectively known as the DNA Damage Response (DDR) [Ciccia and Elledge, 2010]. DNA damage signalling, a key component involved in transduction of the DDR, results from activation of the phosphatidylinositol 3‐kinase‐related kinases (PIKKs) which activate downstream cascades through phosphorylation. Two proteins that are activated downstream in response to DNA damage are Chk1 [Patil et al., 2013] and H2AX [Rogakou et al., 1998]. Recent work in HepG2 cells has identified that both are activated in response to individual PAHs and complex mixtures of PAHs (and other chemicals) found in ambient air and contaminated soil [Mattsson et al., 2009b; Niziolek‐Kierecka et al., 2012; Jarvis et al., 2013]. Activation of H2AX has also been seen previously in A549 cells after exposure to BPDE, the active metabolite of BaP [Mattsson et al., 2009a]. In agreement with these studies we show activation of Chk1 and H2AX in TT1 cells exposed to 3‐NBA and coarse PM samples. Phosphorylation of Chk1 is attributed to the ATR signalling pathway in response to single strand breaks and bulky PAH‐DNA adducts [Jazayeri et al., 2006; Choi et al., 2007, 2009]. High levels of 3‐NBA‐DNA adducts were detected in the TT1 cell line. Whilst there are no previously reported data for 3‐NBA‐DNA adducts in TT1 cells, comparable levels of 3‐NBA‐DNA adducts were seen in this study in A549 cells exposed to 3‐NBA as have been seen previously [Nagy et al., 2005; Arlt et al., 2007]. Levels of BaP‐DNA adducts in TT1 cells were 66‐fold lower than 3‐NBA‐DNA adducts. It is noteworthy that the observed BaP‐DNA adduct levels in TT1 cells are low compared to other published studies [Hockley et al., 2008; Rossner et al., 2016; Shi et al., 2016]. In this study, we showed induction of CYP1A1 in TT1 cells and not a genotoxic response, and hence, this might be reflective of increased repair of BaP‐DNA adducts in the TT1 cell line in the timeframe studied, or a stronger detoxifying effect of CYP1A1; further work is warranted to understand this.

There is extensive epidemiological evidence linking human exposure to PM with adverse health outcomes [Brunekreef and Forsberg, 2005; Kelly and Fussell, 2011; Anderson et al., 2012]. However, the underlying mechanisms behind the exposure to PM and respiratory disease remain elusive. In this study, we exposed cells to coarse and fine PM fractions at 10 µg/ml, which is lower than is commonly used in other studies (typically 50–150 µg/ml and above). Our choice of concentration was informed from other on‐going studies in immortalised and primary human bronchial epithelial cells, dendritic cells and macrophages that have employed concentrations in the range of 0.5–20 µg/ml and that have consistently demonstrated pro‐inflammatory effects and mitochondrial dysfunction at concentrations down to 5–10 µg/ml (unpublished data). The concentration chosen in this study is still relatively high compared to likely human exposures but does provide improved relevance to exposure scenarios occurring in vivo. We showed that in the TT1 cells, exposure to coarse PM (at 10 µg/ml) induced a genotoxic response resulting in increased DNA damage and signalling activation and secretion of IL‐8. Whilst we did not see a comparable response in A549 cells, previous studies have shown that higher concentrations of PM are required to elicit a response [Schins et al., 2002; Danielsen et al., 2008; Thomson et al., 2015], which could explain the lack of response in the present study. A possible explanation for the difference in response of the two cell lines is that the A549 cells are tumour‐derived, and hence they could differ because of genotypic changes. Previously it has been suggested that the A549 cells are an unsatisfactory model of alveolar Type II cells [Swain et al., 2010] and differences in uptake of PM and nanoparticles has been observed between Type I and Type II cells [Kemp et al., 2008].

One interesting observation for the coarse PM data was that the strongest response was seen in the sample from the background location (BG_C). At present the reason for this phenomenon is unclear; total PAH levels in the roadside location were higher (448.20 ng/mg) than those at the background location (200.71 ng/mg) (Supporting Information Table 1). However, it was interesting to note that the levels of the 8 PAHs that were analysed and that have IARC classifications of 1, 2A or 2B were nearly 50% higher in the background sample compared to the roadside (26.14 ng/mg compared to 17.72 ng/mg) (Supporting Information Fig. 1). Whilst this is consistent with the higher genotoxic response seen with the background coarse PM sample, it is important to note that the equivalent PAH concentrations in the fine fraction were markedly higher; 119.01 ng/mg and 43.26 ng/mg at the background and roadside sites, respectively.

The lack of a genotoxic response in cells incubated with fine PM was an unexpected finding and at present the explanation is unclear. Previous studies that have employed samples collected as part of the HEPMEAP project failed to demonstrate that the fine PM fraction was associated with increased inflammatory effects in vitro [Guastadisegni et al., 2010], and there is evidence that the coarse PM was the more pro‐inflammatory in rats treated by intratracheal instillation [Gerlofs‐Nijland et al., 2007]. In both of these studies the overall enhanced pattern of toxicity was associated with the coarse PM samples collected across Europe and across different seasons. It is important to note that the samples used in these studies, as well as here, have a cut off between 0.1 and 2.5 µm. It is therefore plausible that the bioactive element of fine PM could be attributed to the ultrafine (<0.1 µm) particles. Indeed there is growing interest in understanding the adverse health effects of human exposure to ultrafine particles and engineered nanoparticles [Li et al., 2016], which were not present in the PM samples employed in this study.

In conclusion, this study investigated the genotoxic effects of coarse and fine PM from high and low traffic sites in two alveolar epithelial models, the recently described TT1 human alveolar epithelial cell line and the commonly used (tumour‐derived) A549 cells. A genotoxic response was observed in TT1 after exposure to coarse PM that, contrary to expectations, was stronger in cells exposed to PM from a background site compared to the roadside. Furthermore, we showed a strong genotoxic response to 3‐NBA in the TT1 cells that was not seen with BaP. Finally, we found that the TT1 cell line is a more sensitive and improved in vitro model for studying PM toxicity than the A549 cell line.

## Supporting information

Supporting Information
