# Supplementary material for: Genotoxicity of fine and coarse fraction ambient particulate matter in immortalised normal (TT1) and cancer‐derived (A549) alveolar epithelial cells
Source: Environ Mol Mutagen. 2018 Jan 25;59(4):290–301. doi: 10.1002/em.22166 (PMC5947684; doi:10.1002/em.22166)
Supplement: Supplementary file 1 — Supporting Information [file EM-59-290-s001.docx]

**Supplementary Materials**

**Supplementary Table 1.** Overview of the PAH content in the four PM samples used in this study and total mass of PAHs. Values are given in ng/mg.

**Supplementary Table 2.** Overview of the metal content in the four PM samples used in this study and total mass of PAHs. Values are given in µg/mg.

**Supplementary Figure 1.** Compositional details of the coarse and fine PM samples employed in the study from an urban background and roadside site. Panel A illustrates the concentrations of the 8 PAHs analysed in the coarse and fine PM samples that have IARC classifications of 1, 2A or 2B. Masses are given as ng/mg. Panel C the concentrations of the 7 metal/metalloid compounds within the PM samples with IARC classifications. Panel C shows the ascorbate and glutathione dependent oxidative potential (OPAA and OPGSH) per unit mass of PM of the 4 PM samples. Vales represent the mean ±SD of triplicate incubations.

**Supplementary Figure 2.** Determination of cell viability in TT1 cells exposed for 24 hours to 0 – 3.6 µM 3-NBA (A) or 0 – 39.6 µM BaP (B). Cell viability was assessed by AlamarBlue assay. Experiments were performed at least in triplicate and data points represent the mean values ± SD. Data are normalised to control levels (at 100 %) and hence no error bars are present in the control samples.

**Supplementary Figure 3.** DNA adducts formation measured by ^32^P-postlabelling in TT1 cells exposed to3.6 µM 3-NBA(A) or 39.6 µM BaP (B). Autoradiographic profiles of DNA adducts formed after enrichment with butanol extraction (3-NBA) or nuclease P1 (BaP). The origins (OR), at the bottom left-hand corner, were cut off before exposure. N1, 2′(2′-deoxyadenosine-*N^6^*-yl)-3-aminobenzanthrone (dA-*N^6^*-3-ABA); N2, as-yet-unidentified adenine adduct derived from nitroreduction; N3, *N*-(2′-deoxyguanosine-*N^2^*-yl)-3-aminobenzanthrone (dG-*N^2^*-3-ABA); N4, N-(2′-deoxyguanosin-8-yl)-3-aminobenzanthrone (dG-C8-*N*-3-ABA); B1, 10-(deoxyguanosin-*N^2^*-yl)-7,8,9-trihydroxy-7,8,9,10-tetrahydro-BaP (dG-*N^2^*-BPDE).

**Supplementary Table 1**

| **PAH** | **MW** | **Rings** | **Road_C** | **Road_F** | **Urban_C** | **Urban_F** |
| --- | --- | --- | --- | --- | --- | --- |
| Naphthalene | 128.17 | 2 | 2.03 | 3.90 | 1.43 | 1.37 |
| 1-Methylnaphthalene | 142.20 | 2 | 0.37 | 0.00 | 0.28 | 0.39 |
| 2-Methylnaphthalene | 142.20 | 2 | 0.37 | 0.00 | 0.28 | 0.39 |
| 2,6-Dimethylnaphthalene | 156.22 | 2 | 2.01 | 4.09 | 0.39 | 0.27 |
| 2,3,5-Trimethylnaphthalene | 170.25 | 2 | 1.39 | 1.75 | 1.31 | 0.73 |
| Acenaphthylene | 152.19 | 3 | 4.52 | 5.31 | 0.37 | 0.37 |
| Acenaphthene | 154.21 | 3 | 3.42 | 14.68 | 0.14 | 0.39 |
| Fluorene | 166.22 | 3 | 6.79 | 7.28 | 1.39 | 0.58 |
| Phenanthrene | 178.23 | 3 | 101.69 | 31.80 | 40.77 | 14.01 |
| Anthracene | 178.23 | 3 | 2.49 | 2.38 | 1.46 | 0.65 |
| 1-methylphenanthrene | 192.26 | 3 | 5.96 | 3.10 | 26.83 | 9.89 |
| Fluoranthene | 202.25 | 4 | 180.99 | 197.22 | 53.12 | 30.38 |
| Pyrene | 202.25 | 4 | 117.91 | 101.08 | 43.26 | 31.07 |
| Benz[*a*]anthracene | 228.29 | 4 | 3.51 | 5.68 | 5.39 | 18.98 |
| Triphenylene& Chrysene | 228.29 | 4 | 9.18 | 13.69 | 7.50 | 21.37 |
| Benzo[*b*]fluoranthene | 252.31 | 5 | 1.28 | 6.13 | 4.74 | 32.79 |
| Benzo[*k*]fluoranthene | 252.31 | 5 | 0.48 | 5.45 | 1.47 | 8.22 |
| Benzo[*e*]pyrene | 252.31 | 5 | 1.10 | 4.72 | 2.19 | 15.14 |
| Benzo[*a*]pyrene | 252.31 | 5 | 0.52 | 3.62 | 2.06 | 16.58 |
| Perylene | 252.31 | 5 | 0.24 | 1.48 | 0.49 | 3.39 |
| Dibenz[*a,h*]anthracene | 278.35 | 5 | 0.10 | 0.73 | 0.22 | 1.79 |
| Indeno[*1,2,3-cd*]pyrene | 276.33 | 6 | 0.62 | 4.05 | 3.33 | 17.92 |
| Benzo[*ghi*]perylene | 276.33 | 6 | 1.23 | 6.89 | 2.29 | 18.49 |
| **Total** | **-** | **-** | **448.20** | **425.03** | **200.71** | **245.16** |

**Supplementary Table 2**

|  | **Road_C** | **Road_F** | **Urban_C** | **Urban_F** |
| --- | --- | --- | --- | --- |
| **Li** | 0.012 | 0.008 | 0.014 | 0.002 |
| **Be** | 0.001 | 0.001 | 0.001 | 0.001 |
| **Sr** | 0.085 | 0.019 | 0.138 | 0.016 |
| **Mo** | 0.295 | 0.070 | 0.109 | 0.021 |
| **Cd** | 0.003 | 0.007 | 0.004 | 0.010 |
| **Sb** | 0.577 | 0.136 | 0.358 | 0.061 |
| **Ba** | 0.671 | 0.131 | 0.483 | 0.081 |
| **La** | 0.006 | 0.003 | 0.008 | 0.002 |
| **Ce** | 0.016 | 0.006 | 0.016 | 0.004 |
| **Nd** | 0.003 | 0.001 | 0.005 | 0.001 |
| **Sm** | 0.001 | 0.001 | 0.001 | 0.001 |
| **Hf** | 0.129 | 0.049 | 0.066 | 0.010 |
| **Hg** | 0.001 | 0.001 | 0.001 | 0.001 |
| **Tl** | 0.001 | 0.001 | 0.001 | 0.001 |
| **Pb** | 0.234 | 0.267 | 0.191 | 0.368 |
| **U** | 0.000 | 0.000 | 0.001 | 0.000 |
| **Mg** | 9.647 | 1.478 | 12.278 | 0.866 |
| **Al** | 7.646 | 1.761 | 9.734 | 2.635 |
| **Si** | 17.382 | 5.240 | 28.496 | 6.417 |
| **Ca** | 36.179 | 3.223 | 84.496 | 4.496 |
| **Sc** | 0.012 | 0.002 | 0.016 | 0.001 |
| **Ti** | 0.250 | 0.052 | 0.343 | 0.025 |
| **V** | 0.033 | 0.027 | 0.028 | 0.015 |
| **Cr** | 0.275 | 0.061 | 0.114 | 0.051 |
| **Mn** | 1.021 | 0.240 | 0.535 | 0.130 |
| **Fe** | 99.365 | 15.920 | 30.396 | 4.055 |
| **Co** | 0.025 | 0.025 | 0.025 | 0.025 |
| **Ni** | 0.138 | 0.060 | 0.072 | 0.025 |
| **Cu** | 2.386 | 0.457 | 1.456 | 0.264 |
| **Zn** | 1.828 | 1.711 | 3.411 | 2.310 |
| **Na** | 5.916 | 4.205 | 13.293 | 3.584 |
| **K** | 3.321 | 2.413 | 7.498 | 8.705 |
| **As** | 0.251 | 0.251 | 0.251 | 0.251 |
| **Se** | 0.251 | 0.251 | 0.251 | 0.251 |
| **Total** | 187.961 | 38.078 | 194.092 | 34.684 |

**Supplementary Figure 1**

**
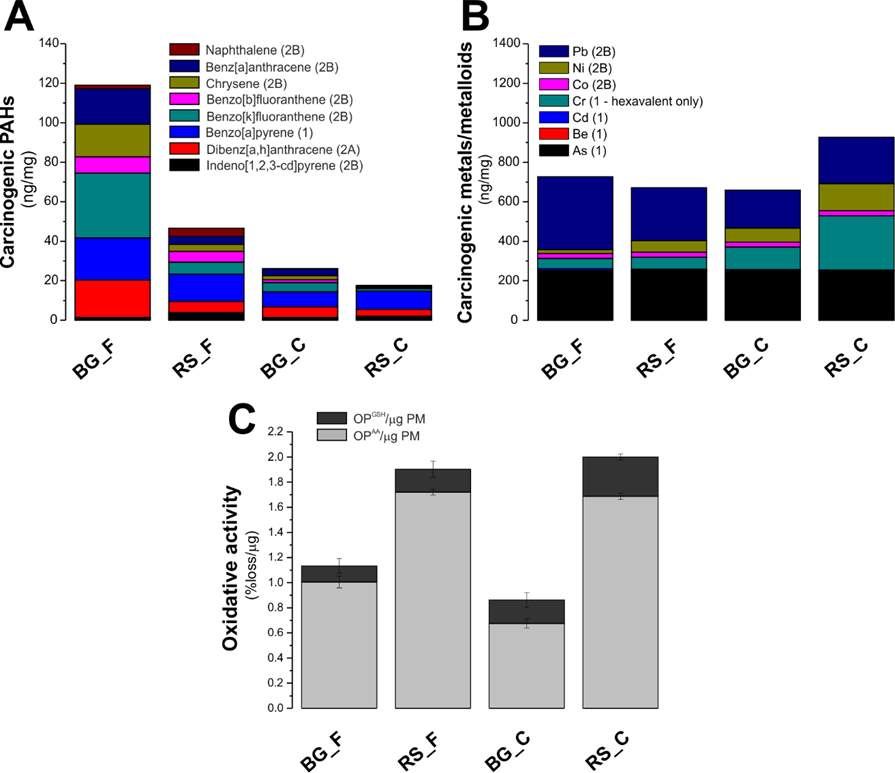
**

**Supplementary Figure 2**


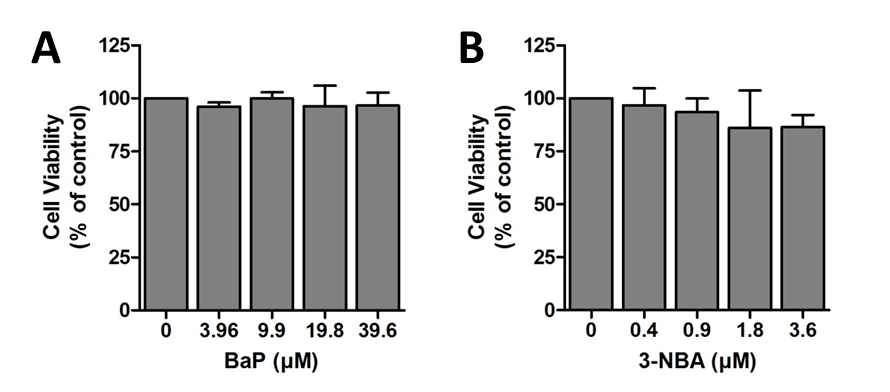


**Supplementary Figure 3**

**
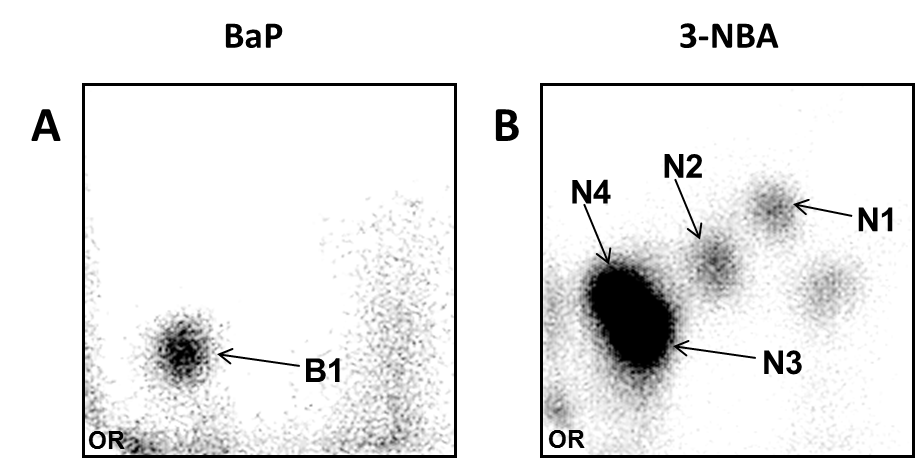
**
